# Molecular Dynamics Simulation of NiTi Shape Memory Alloys Produced by Laser Powder Bed Fusion: Laser Parameters on Phase Transformation Behavior

**DOI:** 10.3390/ma16010409

**Published:** 2023-01-01

**Authors:** Guotai Li, Tianyu Yu, Pan Wu, Mingjun Chen

**Affiliations:** 1 State Key Laboratory of Robotics and System, Harbin Institute of Technology, Harbin 150001, China; 2 School of Mechatronics Engineering, Harbin Institute of Technology, Harbin 150001, China

**Keywords:** molecular dynamics, laser powder bed fusion, NiTi, shape memory alloys, element evaporation, phase transformation

## Abstract

In this study, the deposition, powder spreading, and laser fusion processes during the laser powder bed fusion (L-PBF) process were studied using molecular dynamics (MD) simulation. The effect of Ni content on the characteristic phase transformation temperatures was also investigated. Shape memory effect and superelasticity of NiTi alloys with Ni content ranged from 48.0% to 51.0% were analyzed. By employing MEAM potentials, the effects of the laser power, spot diameter, and scanning speed on the molten pool size and element evaporation were studied. Simulation results showed that a larger spot diameter renders a higher Ni content in the molten pool, also a larger molten pool. A faster scanning speed leads to a higher Ni content in the molten pool, and a smaller molten pool. The element is difficult to evaporate using small laser power and a large spot diameter. The element in the molten pool expresses a great evaporation effect when the Es is larger than 0.4 eV/Å³. According to Ni content within the molten pool during laser fusion, characteristic phase transition temperatures in single crystalline NiTi alloys with variant Ni content were investigated by employing a 2NN-MEAM potential. Characteristic phase transition temperature changes as the Ni content increases from 48.0% to 51.0%. Austenite boundaries and Ni content in the boundary were found to be the keys for controlling the characteristic phase transformation temperature.

## 1. Introduction

Additive manufacturing (AM) of NiTi alloys has been studied in recent years. The relationship between microstructural anisotropy and superelasticity (SE) in large builds fabricated using directed energy deposition (DED) was investigated by Bimber et al. [1]. The Ni_4_Ti_3_ precipitates were observed in the builds. Hamilton et al. [2] studied the multi-scale shape memory effect (SME) in NiTi alloys additive manufactured by L-PBF and DED. The experimental results showed that DED produced NiTi alloys exhibiting a hardening behavior compared to a plateau stress curve for selective laser melting (SLM)-produced counterparts. Furthermore, SME recovery for DED alloys is a fast process, and the initial recovery for SLM alloys requires a finite thermal input. Heterogeneity increases strain within banded localized contours for DED produced alloys. The oriented/detwinned martensite phased were found to be unstable. Wang et al. [3] comparatively studied the in-situ alloying of NiTi alloys by DED, SLM and selective electron beam melting (SEBM) using pre-mixed NiTi powders. The results showed that the printability of pre-mixed NiTi powders using different techniques is ranked as DED > SLM > SEBM. The DED process can better adjust the Ni and Ti ratio, achieving the desired NiTi phase. SLM and SEBM suffered exothermic reactions of the ingredient materials, a lack of fusion and powder-ignition in preheating. In SLM, the change of process parameters could affect the phase transformation performance of final products. Saedi et al. [4] concentrated on the effects of laser power and scanning speeds on the microstructure, transformation temperatures, texture, and shape memory behavior of Ni_50.8_Ti_49.2_ alloys. The energy input plays an important role on the densification of SLM fabricated components. A high laser power should be combined with a high scanning speed and a low laser power with a low scanning speed to fabricate fully dense parts. Wang et al. [5] found that the martensite transformation temperatures change monotonously with the scanning speed, hatch spacing and laser power. The variation of phase transformation temperature is caused by the different amounts of Ni-loss. Meanwhile, Zhou et al. [6] investigated the microstructure, phase transformation behavior and mechanical properties of Ni-rich NiTi alloys fabricated by SEBM. The SEBM-printed NiTi alloy demonstrates excellent and stable SE at room temperature in cyclic compression testing. Its tensile performance was also better than the NiTi alloys produced by SLM.

Ultrashort-laser-induced-plasma on the surface of solid materials before the molten phase has also been widely studied. Penttilä et al. [7] investigated the picosecond laser process, and analyzed the material removal rates of metals. Using high-average-power ultrashort pulse lasers, Schille et al. [8] studied laser processing of technical grade stainless steel and copper to gain deeper insight into material removal for microfabrication. Using picosecond-laser-induced plasma spectroscopy, Fikry et al. [9,10] investigated the effects of laser parameters on the plasma profile of copper and the controlled method of plasma electron number density. Li et al. [11] proposed a hydrochloric acid oxygen assisted femtosecond laser removal process for Cu removal in integrated circuits.

Studies have been conducted experimentally on AM of NiTi alloys. However, atomic-scale simulation of NiTi alloys fabricated by L-PBF is yet to be explored. Molecular dynamics (MD), a common method for atomic-scale simulation, has been used by different researchers to study the thermodynamic properties of different NiTi alloys. Using atomistic modeling, Zhang et al. [12] studied the SE of NiTi SMAs with complex microstructures. Simulation results showed that the obvious stress plateau was only observed in the case of nanocrystalline grains with a tensile strain of 8% and the stress-induced martensitic transformation of NiTi was influenced by grain size, grain orientation, phase composition, substructure, and temperature. Li et al. [13] investigated thermodynamic behavior of NiTi SMAs. Simulation results showed the formation of austenite phase boundaries. Stress arises with the martensitic nucleation, and spreads around with the martensitic phase transition processes. MD simulations were also used to study the mechanical behavior of NiTi alloys under bending [14,15] and nanoindentation [16]. However, limited studies were focused on L-PBF. Wang et al. [17] used MD simulation to study the oxidation of Fe-based powders and found that the L-PBF parameters greatly influenced metal oxidation. Furthermore, metal powder deposition and powder spreading processes have not been well studied.

In this study, we focused on the simulation of L-PBF processes using granulated NiTi powders to simulate pre-alloyed NiTi powder. The vapor pressure has not been considered in the simulation, and only missed atoms were considered instead. It will help to better understand the effect of laser parameters on the element evaporation, melt pool dynamics and Ni content evolution in the molten pool. According to the results of element evaporation in the molten pool during laser fusion, transformation temperatures, SME and SE of NiTi alloys with different Ni content were further analyzed. The L-PBF processes were simulated at first, the granulated NiTi powders were randomly deposited into a NiTi tank. A virtual cylindrical roller was used to compact and spread the NiTi powder. At this stage, the frictional and normal force, and the Von Mises stress were investigated. Then, the effects of laser parameter on NiTi content in the molten pool during the laser fusion process were analyzed. Finally, influence of different NiTi contents on characteristic phase transformation temperatures and the mechanical behavior of NiTi alloys was studied. The outcomes of this study are more qualitative and conceptual than quantitative since the scale for MD simulation is much smaller than the real LPBF process.

## 2. Simulation Processes and Methods

### 2.1. Potential for Molecular Dynamics Simulation of NiTi Alloys

This study used a large-scale atomic/molecular massively parallel simulator (LAMMPS-15Sep2022) [18]. Modified embedded-atom method (MEAM) potentials [19] were used. It is based on the second nearest-neighbor modified embedded-atom method (2NN-MEAM) [20]. The MEAM potentials were calculated with certain melting point, latent heat, and binary phase diagrams of both Ti- and Ni-rich regions. More interatomic potentials were developed for Ni-Ti system, such as the Finnis-Sinclair potentials [21], potential proposed by Lai and Liu [22], Ren and Sehitoglu [23], the modified Finnis-Sinclair potentials [24,25], the embedded-atom method (EAM) potential [26,27], the MEAM potential [28] by Ishida and Hiwatari [29], and other potentials [20,30,31,32,33,34,35]. The MEAM potentials proposed by Kavousi et al. [19] and Ko et al. [20] were more suitable here for laser fusion, and related phase transformations.

### 2.2. Simulation Procedure

#### 2.2.1. The Deposition Process of NiTi Powders

**Figure 1:**
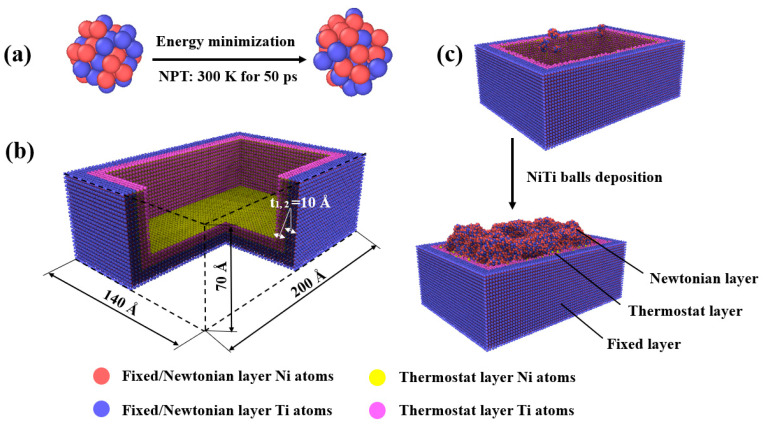
Deposition process: (**a**) preparation of granulated NiTi powder, (**b**) the model of deposition tank, where t_1_ and t_2_ are the wall thickness, and (**c**) granulated NiTi powder deposition process.

Pure granulated Ni powders with a diameter of 9 Å were built at first, and half of the Ni atoms were randomly replaced by Ti atoms as shown in Figure 1a. In order to minimize the energy of the equiatomic granulated NiTi powders, the conjugate gradient method [36] was adopted. Then, the granulated NiTi powders were relaxed at 300 K for 50 ps using the NPT ensemble, where the pressure and temperature were controlled by the Parrinello-Rahman barostat [37] and the Nose-Hoover thermostat [38] method. The timestep used in the simulation was 1 fs. For deposition simulation, a NiTi square tank was built with an outer dimension of 140 × 200 × 70 Å^3^ and an inner size of 100 × 160 × 50 Å^3^ (Figure 1b). The lattice constant of the tank was set as 3.016 Å [20]. The x, y, and z axes correspond to [100], [010], and [001] crystallographic orientations, respectively. The tank consists of 86,295 atoms with red and yellow atoms representing nickel atoms, blue and pink atoms representing titanium atoms. Fixed boundary conditions were applied in all three dimensions. The thermostat layer was relaxed at 300 K for 150 ps using the NVT ensemble. Furthermore, to simulate the deposition process, 1000 granulated NiTi particles were deposited in 500 ps in the z direction at a velocity of −10 Å/ps, red atoms represent deposited nickel atoms and blue atoms represent deposited titanium atoms. The model was then relaxed for 100 ps to reach the equilibrium state. It can be seen from Figure 1c that the model was divided into three layers, where the NiTi tank was divided into a fixed layer and a thermostat layer. The deposited particles were set as a Newtonian layer. The force and the atom velocity of the fixed layer were set as zero. The temperature of the thermostat layer was kept at 300 K by the NVT ensemble. The NVE ensemble was used to maintain energy conservation for the system. The reflect wall was fixed at the height of 105 Å in the z direction to prevent deposited balls flying out from the top.

#### 2.2.2. NiTi Powder Spreading Process

**Figure 2:**
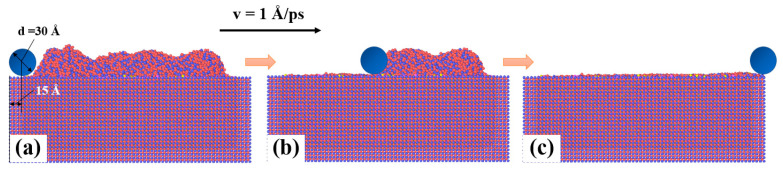
Powder spreading process: (**a**–**c**) the beginning stage, the state at 70 ps, and the end stage, respectively.

Figure 2 displays the powder spreading process, and the model was divided into three parts, as in Section 2.2.1. The boundary conditions used were the same as with the deposition process, and fixed boundary conditions were used in all three directions. The temperature control methods used in each layer were the same as in Section 2.2.1. Virtual cylindrical roller was used for simulation as shown in the Figure 2 (the dark blue ball), and the force between the virtual cylindrical and powders was given by the following repulsive force model:

(1) $$F\left(r\right)=\left\{\begin{array}{c} -K{\left(r-R\right)}^{2},\;r<R\\ 0,\;r\geq R \end{array}\right.$$

where F is the repulsive force, K is the specified force constant, r is the distance from the atom to the center axis of the cylinder and R is the radius of the indenter. K value was set as 10 eV/Å^3^ here [39]. The roller extends infinitely along x axis and moved at a velocity of 1 Å/ps along y axis. The height of center axis of the virtual roller at z axis was 85 Å, and the R was set as 15 Å.

OVITO [40] is used for the analysis of microstructures during the powder spreading process. The Von Mises stress was calculated by the following function:

(2) $$\sigma_{i}^{Mises}=\sqrt{\frac{1}{2}\left[{\left(\sigma_{xx}-\sigma_{yy}\right)}^{2}+{\left(\sigma_{yy}-\sigma_{zz}\right)}^{2}+{\left(\sigma_{xx}-\sigma_{zz}\right)}^{2}+6\left(\sigma_{xy}^{2}+\sigma_{yz}^{2}+\sigma_{xz}^{2}\right)\right]}$$

#### 2.2.3. Laser Fusion Process

**Figure 3:**
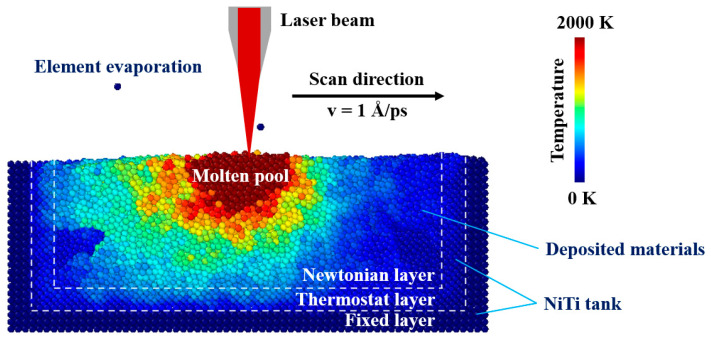
Laser powder bed fusion process simulated using MD models.

In order to better simulate the laser fusion process, the model obtained from the roll process was relaxed at first. The model was divided into a fixed layer, a thermostat layer and a Newtonian layer. The atoms in the fixed layer were fixed. The atoms in the thermostat layer and Newtonian layer were kept at 300 K for 100 ps using the NPT ensemble. The fixed boundary conditions were used in all three dimensions. Then, the temperature of the thermostat layer was controlled at 300 K using the NVT ensemble and the Newtonian layer was controlled by the NVE ensemble, maintaining energy conservation. Non-translational kinetic energy/heat was added to the surrounding atoms through the laser spot. The laser energy density followed a Gaussian distribution. The spot radius, scan speed and laser power were set to 10 Å, 1 Å/ps, and 180 eV/ps. The laser fusion process was shown in Figure 3.

#### 2.2.4. Phase Transformation of NiTi Alloys

**Figure 4:**
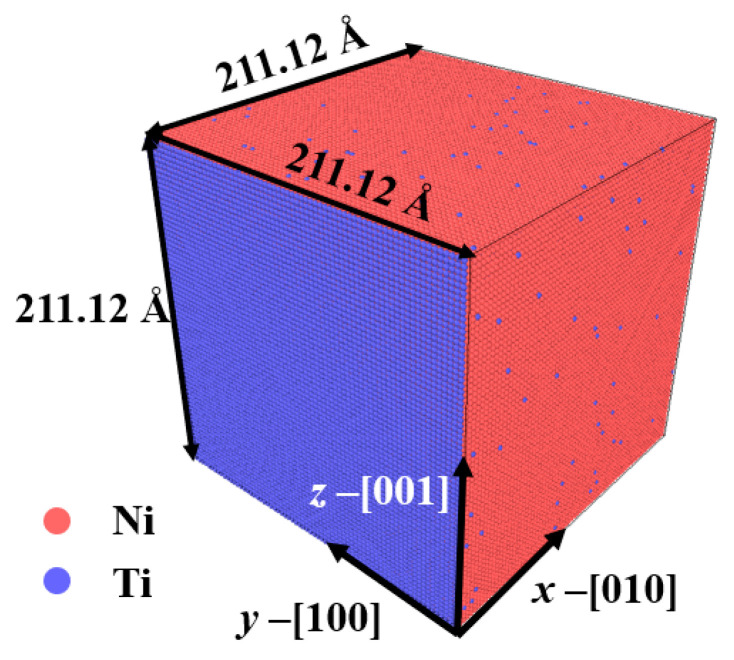
Atomic configuration of a single crystalline NiTi box with 50.5% Ti content.

We constructed [100]-oriented single crystalline NiTi alloys with a dimension of 211.12 × 211.12 × 211.12 Å^3^ was at 600 K, a temperature where phase structure is the B2 (austenite) phase. Certain percentages of Ti atoms were added with different Ti contents. A supercell of NiTi alloys with 50.5% Ti content are shown in Figure 4, where the x, y, and z axes are in [100], [010], and [001], respectively. A supercell consists of 686,000 atoms (red atoms—nickel, blue atoms—titanium). Periodic boundary conditions were applied in all directions. Furthermore, the model was fully relaxed by energy minimization and temperature controlled at 600 K for 150 ps using the NPT ensemble. To study the thermally induced martensitic transformation, the single crystalline model’s temperature was gradually decreased to 50 K from 600 K, and then heated back to 600 K with a cooling/heating rate of 5 K/ps.

## 3. Results and Discussion

### 3.1. The Study of Powder Deposition and Rolling Process

#### 3.1.1. Adsorption Behavior during the Deposition of NiTi Powders

**Figure 5:**
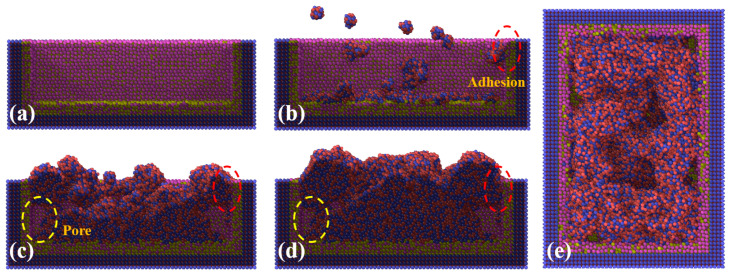
The deposition process of granulated NiTi powders (**a**–**d**) cross-section views corresponding to the state of 0 ps, 50 ps, 300 ps, and 500 ps, respectively. Red circles present adhesion phenomenon. Yellow circles are the pores formed during the deposition process. (**e**) Top view obtained after relaxation.

**Figure 6:**
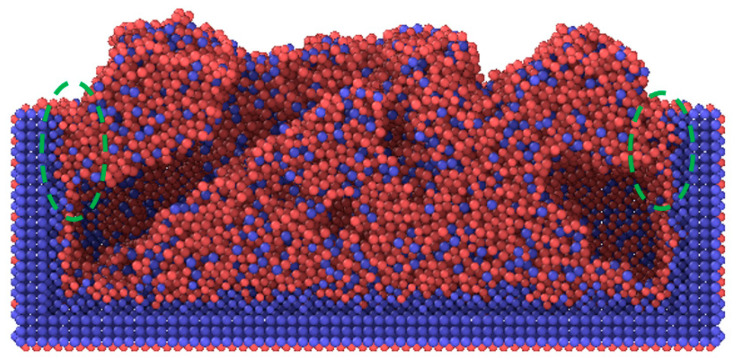
Phase of the deposited material showing austenite (blue) and martensite (red), respectively. The green dotted circles reflect the wall-adhesion region.

Powder deposition, as the first step of the LPBF process, has an important influence on the thermal and mechanical behaviors of the NiTi alloys in the following processes. To investigate the L-PBF process systematically on an atomic scale, the powder deposition process was first studied as shown in Figure 5. The granulated NiTi powders were deposited at the bottom of the tank and part of the powders adhered to the inside walls of the tank. As the powders continued being deposited, pores could be formed under a combined effect of powder accumulation and adhesion, marked by red and yellow circles in Figure 5. Furthermore, Figure 5e displays the top view of the deposition model after relaxation. It can be observed that the surface flatness of the deposited powders is poor. It was found that decreasing the size of granulated NiTi powders or increasing the deposition speed are beneficial to the improvement of deposition surface roughness. Figure 6 shows the phase of the deposited material, under collision between the granulated powder and deposition tank, the phase of powder changes from austenite to martensite. It can be seen from the green circles (Figure 6) that martensitic phase transformation occurred on the inner wall of the tank during adhesion.

#### 3.1.2. Dynamic Evolution and Force Analysis of NiTi Alloys during Spreading Process

**Figure 7:**
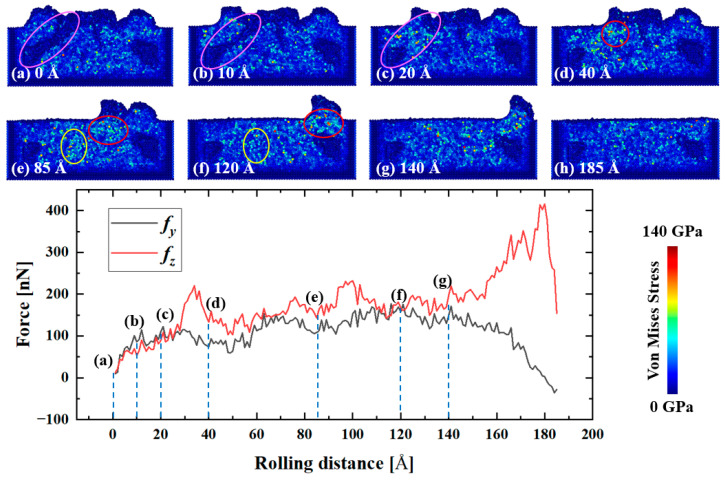
Microstructural evolution and the force variation with rolling distance during the rolling process, where (**a**–**h**) represent the cross-section view of the MD model at different rolling distances. *fy* and *fz* represent the frictional and the normal force. The pink, red, and yellow circles track the internal stress evolution in these regions during the rolling process.

The deposition procedure in Section 3.1.1 is followed by the powder spreading process prior to the laser fusion process. As in L-PBF, the powder spreading process is used to flatten the powder bed, making powders uniformly and compactly distributed, suppressing the formation of defects. Figure 7 shows the microstructural evolution and force curves with rolling distance during the powder spreading process. It can be found that internal stresses still exist in the deposition model after relaxation by analyzing the Von Mises stress diagram as shown in Figure 7a. The powder adhered to the inner walls of the tank was able to fill the internal pores left (pink circle in Figure 7). A large force was generated when the adhered atoms were pressed against the deposited atoms, showing a significant increase in the Von Mises stress (pink circle in Figure 7c) and normal force (100 nN increased to 200 nN as shown in the force curves in Figure 7 between the point c and d). Further analysis of the Von Mises stress shows that the stress was mainly concentrated in the lower front of the roller (the red circle). When the roller passed away, the internal stress decreased under the influence of the thermostatic layer. In general, the frictional and normal forces were relatively stable during the powder spreading process.

### 3.2. Effects of Laser Parameters on Elemental Content in Molten Pool during L-PBF

As the key parameters during the L-PBF process, the laser power, spot diameter, and scanning speed were analyzed in this study to investigate their influence on the molten pool size and Ni evaporation amount. The process parameters used in the laser fusion simulation process are shown in Table 1. The phase of deposited materials was a mixture of martensite and austenite before laser irradiation, where martensite was formed by SME of NiTi alloys under stress. Under a high temperature induced by laser irradiation, part of the martensite transformed to austenite, but the temperature in the phase transition region decreased rapidly with the departure of the laser source. Due to the internal stress, part of the austenite phase transformed back to the martensite phase. Single scanning lines were generated by irradiating the NiTi model at a laser power from 120 to 240 eV/ps with a spot diameter of 15 Å, 20 Å, and 25 Å. The scanning speed from 0.5 to 5 Å/ps were studied at a laser power of 180 eV/ps and a spot diameter of 20 Å.

The effect of the laser power and scanning speed on the laser fusion process can be attributed to energy density (Ed) input [41,42], which represents the energy transferred to a certain area and is calculated by the following equation:

(3) $$E_{d}=\frac{P}{V_{s}\cdot h\cdot d}$$

where the *P* represents the laser power, *V_s_* is the laser scanning speed, *h* is the hatch space, and *d* is layer thickness. In this study, a single-layer with single pass scanning was used. Energy can be calculated by the following equation:

(4) $$E_{s}=\frac{P}{V_{s}\cdot \pi \cdot r^{2}}$$

where the *E_s_* is the energy input per unit area, *r* is the laser spot radius. From Equation (4), it can be found that a larger spot radius will lead a smaller energy input per unit area.

**Figure 8:**
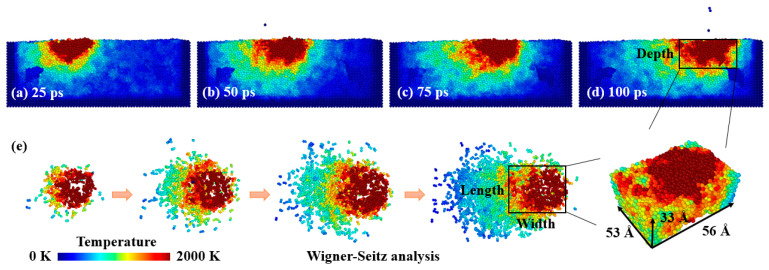
The molten pool evolution and the Wigner-Seitz analysis [45] during L-PBF with a laser power of 180 ev/ps, a spot diameter of 20 Å, and a scanning speed of 1 Å/ps: (**a**–**d**) are the cross-section view at time of 25 ps, 50 ps, 75 ps, and 100 ps, respectively; (**e**) molten pool morphology evolution.

The molten pool evolution was analyzed for the model with a laser power of 180 ev/ps, a spot diameter of 20 Å, and a scanning speed of 1 Å/ps as displayed in Figure 8. The pre-deposited NiTi powder was melted with the irradiation of the laser beam. A temperature of 2000 K was selected as the upper limit temperature in the molten pool according to the experimental and simulation results obtained by Watanabe et al. [43] and Kavousi et al. [19]. The Wigner-Seitz analysis in Figure 8 displays that the molten pool size increased with the movement of the laser beam. With the continuous laser energy input, the temperature dropped rapidly after the laser beam passed by. A large thermal gradient and fast cooling rate were confirmed [44].

**Figure 9:**
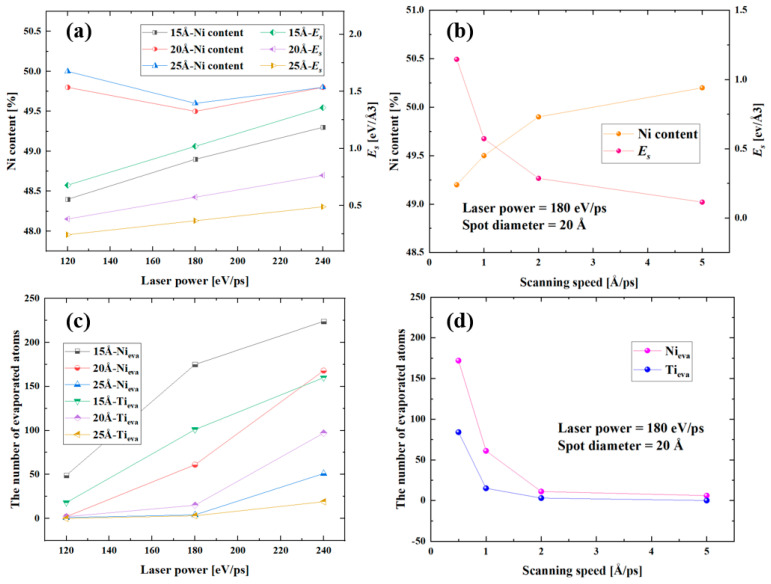
The influence of laser parameters on the Ni content in the molten pool and *E_s_*: (**a**) the effect of laser power and spot diameters on the Ni content and *E_s_* at the same scanning speed of 1 Å/ps. (**b**) the effect of the scanning speed on the Ni content and *E_s_* at the laser power of 180 eV/ps and a spot diameter of 20 Å. (**c**,**d**) the number of evaporated atoms correspond to (**a**,**b**), respectively.

The effect of the laser parameters on the molten pool size and Ni content within the molten pool were investigated. The influence of the laser parameters on the Ni content in the molten pool and Es are shown in Figure 9. It can be seen from Figure 9a that a larger laser spot diameter leads to a lower Es, and a higher Ni content in the molten pool. Furthermore, a larger laser spot diameter leads to a smaller number of evaporated Ni and Ti atoms and a larger molten pool, as shown in Table 1, which explains the higher Ni content in the molten pool. Taking a laser power of 180 ev/ps and a scanning speed of 1 Å/ps as an example, when the laser spot diameter increased from 15 Å to 25 Å, the molten pool size changed from 46 × 41 × 29 Å³ to the 53 × 56 × 33 Å³, and then to a size of 55 × 56 × 33 Å³. The number of evaporated Ni and Ti atoms decreased from 175 to 4, and from 101 to 3, respectively. The Ni content in the molten pool increased from 48.9% to 49.5%, and to 49.6%. Figure 9b shows that a higher scanning speed leads to a higher Ni content in the molten pool. Also, there are fewer evaporated Ni and Ti atoms as shown in Table 1. Figure 9c,d display the number of evaporated atoms corresponding to Figure 9a,b, respectively. It is worth noting that when the spot diameter and scanning speed were the same, an increase in laser power did not influence the Ni content monotonically as shown in Figure 9a. To explain this phenomenon, the number of evaporated Ni and Ti atoms were analyzed. As shown in Figure 9c, both the number of evaporated Ni and Ti atoms increased with the laser power when the spot diameter and the scanning speed are the same. However, the number of evaporated Ni and Ti atoms was almost zero when the laser power was 120 eV/ps and a spot diameter larger than 20 Å, which means that the element was difficult to evaporate when the laser power was small with a large spot diameter, and the molten pool showed a high Ni content. According to the calculation of *E_s_*, the element in the molten pool expressed a great evaporation effect when the *E_s_* was larger than 0.4 eV/Å³.

### 3.3. Influence of Different Element Content on the Phase Transformation Behavior

**Figure 10:**
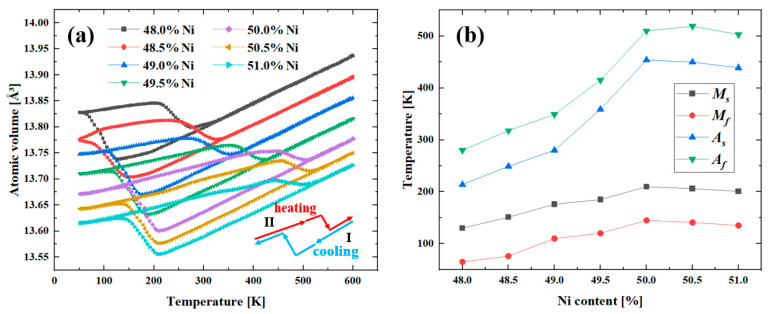
(**a**) The specimen volume for single crystalline NiTi alloys with different Ni content during cooling and heating processes. (**b**) Phase transformation temperature for single crystalline NiTi alloys with different Ni content.

**Figure 11:**
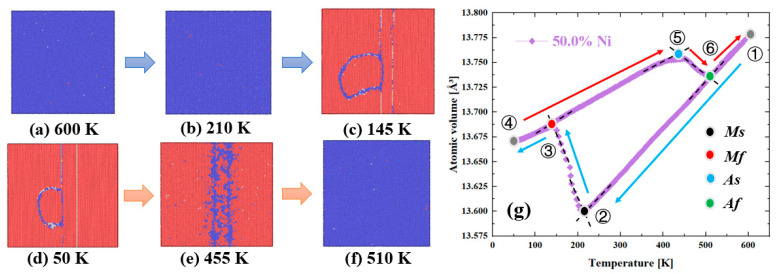
(**a**–**f**) Phase structure of equiatomic NiTi model during cooling and heating processes. The blue and red atoms represent austenite and martensite structures, respectively. (**g**) Evolution of atomic volume during cooling and heating processes, where ①–⑥ correspond to (**a**–**f**).

Phase transformation temperatures typically are martensite start temperature (Ms), martensite finish temperature (Mf), austenite start temperature (As), and austenite finish temperature (Af) for NiTi SMAs. The phase transformation temperature of single crystalline NiTi with 48.0% to 51.0% Ni content was investigated. The Ni content was affected by elemental evaporation during the L-PBF process. Figure 10a shows the volume evolution during cooling and heating processes. Ms increased from 130 K to 210 K and dropped to 201 K for 48.0% to 51.0% Ni content. Mf increased from 65 K to 145 K, and then decreased to 135 K. Compared with Ms and Mf, Ni content exhibited stronger effects on As and Af, which increased from 214 K to 454 K, and decreased to 439 K, increased from 280 K to 519 K, and decreased to 503 K, respectively. The characteristic phase transformation temperature reached the highest for the models with 50.0% Ni content, which is consistent with experimental study [46]. The characteristic phase transformation temperature was determined by the evolution of phase structure and atomic volume. Figure 11 displays the phase structure of the equiatomic NiTi model during cooling and heating processes. When the temperature decreased from 600 K to 210 K, the model mainly consisted of austenite. Martensitic phase transformation occurred when the temperature dropped. Martensitic phase transformation finished at the temperature of 145 K and the size of the remaining austenite phase boundary decreased with the temperature drop to 50 K. The reverse martensitic phase transformation occurred during the heating process near the austenite phase boundary at first. When the temperature increased to 510 K, the reverse martensitic phase transformation finished and the single crystalline structure consisted of austenite.

**Figure 12:**
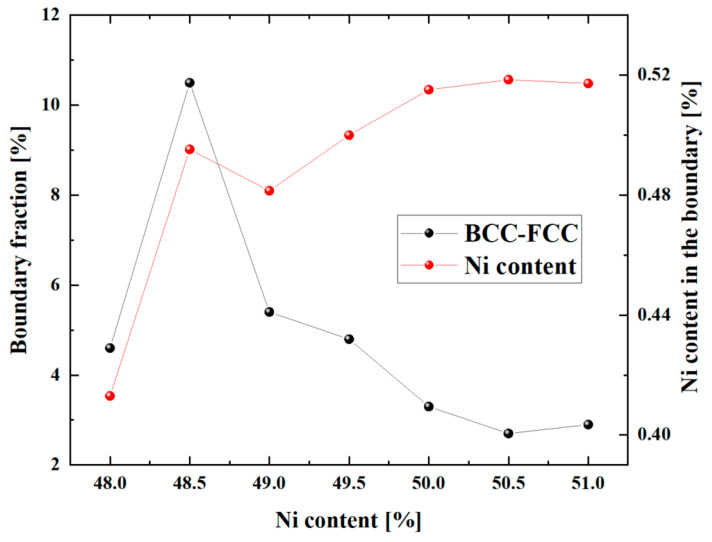
Variation of the BCC-FCC boundary volume fraction and Ni content in the boundary with global Ni content ranges from 48.0% to 51.0% at a temperature of 50 K.

**Figure 13:**
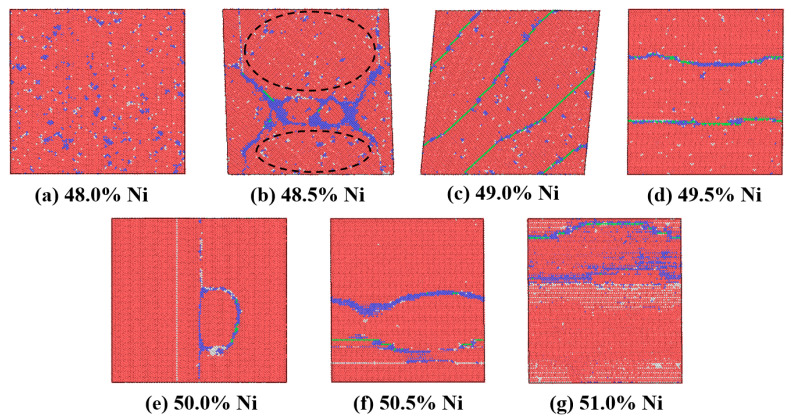
Microstructures with different Ni content ranging from 48.0% to 51.0% at a temperature of 50 K, where red, blue, green and white atoms represent the HCP, BCC, FCC and other structures, respectively.

The influence of Ni content on the phase transformation temperature of single crystalline NiTi was studied. The content of the phase boundary which consists of BCC and FCC structures was analyzed. From Figure 12, the phase boundary content with Ni content ranges from 49.0% to 51.0% decreases from 5.4% to 2.7%, then changes to 2.9%, which was inverse to the trend of the characteristic phase transformation temperature. In addition, the Ni content within the boundary for Ni content from 49.0% to 51.0% better reflects the variation of the characteristic phase transformation temperature. It was worth noting that the phase boundary content and Ni content in the boundary did not reflect the characteristic phase transformation temperature when the Ni content was less than 49.0% for the sample. In addition, the microstructures with different Ni content ranges from 48.0% to 51.0% at the temperature of 50 K were analyzed. It can be found from Figure 13 that the austenite boundary structure exists in the single crystalline NiTi when the Ni content is larger than 48.5%. The austenite boundary becomes unstable in the microstructure with 48.5% Ni content as shown in the Figure 13b marked by the black dashed oval, and the austenite boundary structure did not exist in the model with Ni content of 48.0%.

In summary, the variation of the austenite boundary content and Ni content within the boundary for different Ni content account for the change of the characteristic phase transformation temperature. The austenite boundary gradually disappeared in the single crystalline NiTi when the Ni content was less than 48.5%. This inhibiting effect of the austenite boundary on the martensitic phase transformation is similar to Ni_4_Ti_3_ precipitates, which formed during martensitic transformation, suppressing the martensitic phase transformation [13,47].

**Figure 14:**
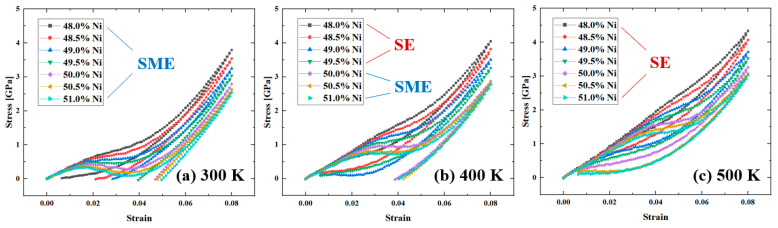
Stress-strain of single crystalline NiTi specimen with different Ni content under compression at the temperature of (**a**) 300 K, (**b**) 400 K and (**c**) 500 K.

Different temperature such as 300 K, 400 K, and 500 K were selected to investigate the SME and SE of single crystalline NiTi alloys with different Ni content according to the Af shown in Figure 10. As shown in Figure 14, increasing Ni content leads to drops in the critical phase transformation stress. Higher Ni content leads to more obvious stress hysteresis. The single crystalline NiTi with different Ni content shows different mechanical properties at 400 K, the Ti-rich model showing SE, and the NiTi with Ni content ranged from 50.0% to 51.0% shows SME. Ni content variation affects the characteristic phase transformation temperature. The specific phase transformation process, SME and SE behavior of single- and nano-crystalline NiTi can be found elsewhere [13,48,49].

## 4. Conclusions

The deposition, powder spreading, and laser fusion process during the L-PBF process were studied using molecular dynamics simulation. The effect of Ni content changes on the characteristic phase transformation temperature was also investigated. The influence of laser parameters on the molten pool size and the Ni content in the molten pool were investigated, since Ni and Ti atoms can evaporate during melting. According to the variation of Ni content in the molten pool during laser fusion, the characteristic phase transformation temperature in the single crystalline NiTi with different Ni content ranges from 48.0% to 51.0% were investigated. Major findings in this work are summarized as follows:

- Adsorption and accumulation of granulated NiTi powders occur during the deposition process. Increasing the powder deposition rate and reducing the granulated powder size could reduce pore size. Internal stresses are present in the deposited atoms after deposition.
- Rolling processes can reduce the number of micro-pores and defects. The friction is stable during the rolling process. The interaction of the adsorbed atoms with the deposited atoms leads to a significant increase in normal force under the action of the roller.
- The effects of laser power, spot diameter, and scanning speed on the molten pool size and element evaporation were studied. A larger spot diameter and a lower *E_s_* leads to a higher Ni content in the molten pool. A faster scanning speed and a lower *E_s_* leads to a higher Ni content in the molten pool.
- The Ni or Ti element is difficult to evaporate when the laser power is small and laser spot diameter is large. The element in the molten pool expresses a great evaporation effect when the *E_s_* is larger than a threshold of 0.4 eV/Å³.
- The characteristic phase transformation temperature increases and then decreases as the Ni content changes from 48.0% to 51.0%. The variation of the austenite boundary content and the Ni content within the boundary for different Ni content account for the change of the characteristic phase transformation temperature.

## Figures and Tables

**Table 1:** The molten pool size, Ni content, and number of evaporated atoms at different process parameters used in the simulation.

No.	Laser Power (eV/ps)	Spot Diameter (Å)	Scanning Speed (Å/ps)	Molten Pool Size (Å³)	Ni content (at. %)	Ni_eva_^a^	Ti_eva_^b^
1	120	15	1	40 × 38 × 20	48.4	49	18
2	120	20	1	40 × 38 × 25	49.8	2	2
3	120	25	1	44 × 35 × 25	50.0	1	0
4	180	15	1	46 × 41 × 29	48.9	175	101
5	180	20	0.5	58 × 60 × 35	49.2	172	84
6	180	20	1	53 × 56 × 33	49.5	61	15
7	180	20	2	51 × 52 × 25	49.9	11	3
8	180	20	5	35 × 50 × 20	50.2	6	0
9	180	25	1	55 × 56 × 33	49.6	4	3
10	240	15	1	51 × 52 × 35	49.3	224	160
11	240	20	1	58 × 68 × 35	49.8	168	97
12	240	25	1	60 × 77 × 35	49.8	51	19

^a^ The number of evaporated Ni atoms; ^b^ The number of evaporated Ti atoms.

## Data Availability

Not applicable.
